# Risk of hematological malignancies in the families of patients treated for nodular lymphocyte-predominant Hodgkin lymphoma

**DOI:** 10.1186/s13053-021-00175-0

**Published:** 2021-02-09

**Authors:** Saad Akhtar, M. Shahzad Rauf, Amani Al-Kofide, Mahmoud A. Elshenawy, Ali Hassan Mushtaq, Irfan Maghfoor

**Affiliations:** 1 King Faisal Specialist Hospital and Research Center, Oncology Center, P.O. Box 3354, MBC# 64, Riyadh, 11211 Kingdom of Saudi Arabia; 2 Department of Pediatric Hematology/Oncology, King Faisal Specialist Hospital and Research Center, Riyadh, 11211 Kingdom of Saudi Arabia; 3 College of Medicine, AlFaisal University, Riyadh, Kingdom of Saudi Arabia

**Keywords:** Hodgkin lymphoma, Nodular lymphocyte-predominant Hodgkin lymphoma, Familial hematological malignancy, Hereditary malignancy, Familial lymphoma, Familial malignancy

## Abstract

**Background:**

Familial clustering of lymphoid and/or hematological malignancies (FHM) provides an opportunity to study the responsible genes. The data is limited in patients with lymphoid and hematological malignancies.

**Methods:**

The lymphoma database was used to identify patients seen in our institution from 1998 to 2019 with nodular lymphocyte-predominant Hodgkin lymphoma (NLPHL). We studied FHM by collecting detailed history of any malignancy in the family (FM).

**Results:**

Two hundred NLPHL patients were identified. Contacting was not possible in 30 patients due to no response to the phone calls (22) and death [1]. 170/200 patients were interviewed; represented 167 families (3 patients with a family member with NLPHL). These 170 patients provided information about 8225 family members. These 167 families had a total of 329 family members with 334 malignancies (including 167 NLPHL patients and 5 members with 2 malignancies each). Of these 167 patients, 77 (46.1%) had no FM while 90 (53.9%) patients had a positive FM; 162 family members with 167 malignancies. Among these 167 families, 31 families (18.6%) had members with FHM +/− solid cancers. These 31 families had 35 family members (25 males:10 females) with 16 lymphomas: diffuse large B cell lymphoma [2], follicular center cell lymphoma [3], chronic lymphocytic leukemia/small lymphocytic lymphoma [3], non-Hodgkin lymphoma [2], classical HL [2], and NLPHL [4]. Total of 8 leukemia: acute lymphoblastic leukemia [4], acute myeloid leukemia [3], and leukemia - no subtyping [5]. These 35 FHM members are 1st [6], 2nd (16), and 3rd [7] degree relatives of 31 NLPHL patients. There are 4 families with NLPHL in family members; all these 8 NLPHL patients are male and are alive. The median total number of 1st + 2nd +3rd degree members are 81. The decrease in the age of diagnosis from 1st generation to the 2nd generation (anticipation) was noted in 13/17 patients; 2nd generation median age at diagnosis was 29.7 years vs 1st generation age 53 years (developed malignancy 23.3 years earlier).

**Conclusion:**

FHM is frequent in NLPHL. This study provided us many important insights for planning future studies in terms of interviewing technique, time, and resource allocation and genetic testing.

**Supplementary Information:**

The online version contains supplementary material available at 10.1186/s13053-021-00175-0.

## Introduction

There is emerging data indicating the role of inheritance in the development of various malignancies. Familial clustering of lymphoid and/or hematological malignancies (FHM) like non-Hodgkin lymphoma (NHL), Hodgkin lymphoma (HL), multiple myeloma (MM), various leukemia and chronic lymphocytic leukemia/small lymphocytic lymphoma (CLL/SLL) have been reported [1–9]. There is limited literature on FHM in patients with nodular lymphocyte-predominant Hodgkin lymphoma (NLPHL) [10–14]. FHM provides an opportunity to study genetic and environmental factors as a causative agent for these conditions and may help in identifying the responsible genes. Almost all the data on FHM are coming from North American and European countries with smaller family sizes. Middle Eastern social setup and population is significantly different from American and European setup. Impact on FHM of large family size, tribal lifestyle, social, cultural environmental factors, consanguinity marriages, and marriages within the tribe are largely unknown and may have contributing effects. This study is to identify FHM in patients with NLPHL seen in our Medical Oncology Lymphoma clinics and to establish a FHM database in the Oncology Research Unit. We intend to use this data for future planning of comprehensive data collection, genetic counseling, genetic studies, and tissue banking.

## Methods

Institutional Research Advisory Committee and Ethics Committee approved the comprehensive data collection study (Clinical Trials.gov Identifier: NCT00538551) for lymphoma patients and the supplemental questionnaire of family history of malignancy (FM) case report form (FM-CRF). FM-CRF was designed to capture all solid cancers and FHM in 1st, 2nd, and 3rd degree relatives, consanguinity marriages, age at diagnosis, treatment, and survival status. Information regarding half brothers and sisters (not an uncommon finding) was also captured. Another form was designed in Arabic, Supplementary Data 1, for data capturing by three native Saudi Arabic speaking oncology nurses with the help of patients. The questionnaire was administered in person to the patient and/or their parents/caregivers in the outpatient routine clinic visit.

Limited discharged patients were contacted via telephone. All the questions were asked even if the patient initially denied/reported any malignancy. Relatives treated at our institution were verified using the hospital medical record and/or Hospital Tumor Registry (data from 1974 to 2014). Degree of the relation is defined as 1st degree relatives (parents, siblings, or child), 2nd degree relatives (uncle, aunt, nephew, niece, grandparent, grandchild or half-sibling/step brother or sister) and 3rd degree relatives (first cousin, great-grandparent or great-grandchild). Confirmed FM required any one of the following; a. a pathology report, b. medical report with the diagnosis, c. reliable history with confirmation of malignant disease by a healthcare professional, d. confirmation from The Saudi Cancer Registry (site office in our Research Unit, data available from 01 January 1994–2015). Saudi Cancer Registry Saudi Arabia is a population-based registry under the jurisdiction of the Ministry of Health. If the patient’s FM was not confirmed by the above a,b,c,d criteria, it was captured as an “unconfirmed case”, for future verification (next phase). The data collection is ongoing and due to significant difficulties and time required for the complete project, we limited the initial “verification of collected data” as a pilot project in NLPHL patients. Also, confirmations of malignancies were limited to FHM.

## Results

**Fig. 1:**
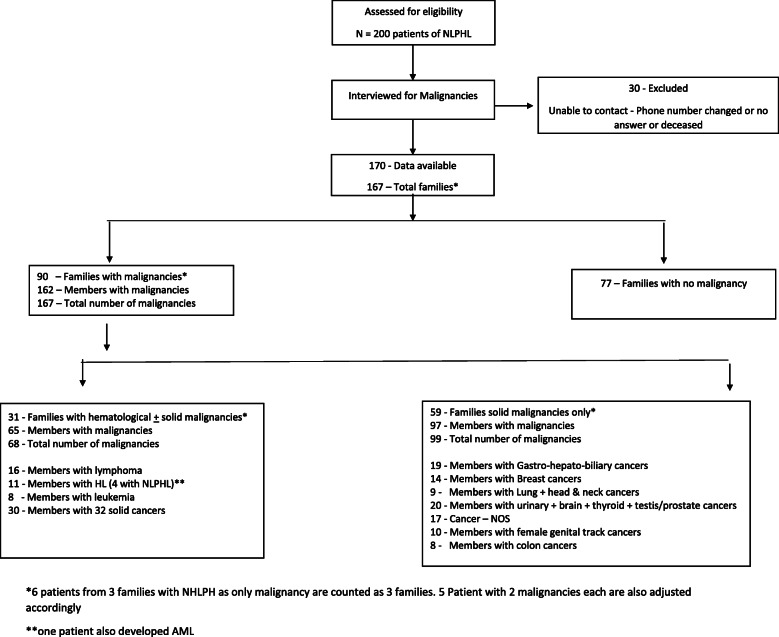
Patient’s selection and distribution of various malignancies

From 1998 to July 2019, 2 hundred NLPHL patients were identified. The results and the selection process are shown in Table 1 and Fig. 1. Contacting was not possible in 30 patients due to the upgrading of the national telephone system (22) and death (8). FM-CRF was available for 170/200 patients, representing 167 families (3 patients have other family members with NLPHL in the data).

**Table 1:** Patient’s characteristics and outcome

Variable	Total numbers	Percentage
Patients with NLPHL	200	100
No data available	30	15
Malignancy related data available	170	65
Total families* of 170 patients	167	–
Male	130	76.5
Female	40	23.5
Median age	20.9 years	Range (5–64)
Age < 21	86	50.6
Type and frequency of familial malignancies		
Family with no malignancy	77	46.1
Families with malignancy	90	53.9
Families with hematological only*	14	8.4
Families with hematological + solid	17	10.2
Families with solid cancers only	59	35.3
Total malignancies in family members	167	–
Total members with malignancy	162	–
Malignancy confirmed	73	43.7
Confirmed among 35 hematological**	28	80
Confirmed among 135 solid**	45	33.3
Malignancy unconfirmed	94	56.3
Unconfirmed among 35 hematological**	7	20
Unconfirmed among 135 solid**	90	66.66
First degree members	51	31.5
Second degree members	85	52.5
Third degree members	26	16
Common familial malignancy by groups*		
Hematological malignancies		
Non-Hodgkin lymphoma	16	9.6
Hodgkin lymphoma	11	6.6
Leukemia	8	4.8
Solid malignancies		
Gastrointestinal	36	21.6
Female genital	13	7.8
Breast	18	10.77
Cancer not otherwise specified	19	11.4
Lung / Head and Neck	17	10.2
Others	27	16.17

Percentages are counted for 170 available patients

*6 patients from 3 families with NHLPH as the only malignancy are counted as 3 families

**Percentages among the hematological and solid malignancies

5 Patient with 2 malignancies each are also adjusted accordingly

Overall results of the entire cohort:

In the entire cohort of 167 patients, 77 patients (46.1%) had no FM while 90 patients (53.9%) had a positive FM; 162 family members with 167 malignancies. Among these 167 families, 31 families (18.6%) had members with FHM +/− solid cancers and, 59 families had members with solid malignancies. These 167 families, in total, had 329 family members with 334 malignancies (including 167 NLPHL patients and 5 members with 2 malignancies each). The current detailed analysis was limited to FHM.

### Hematological malignancies

In these 31 families with FHM, there were 35 family members (25 males:10 females) with various lymphomas and hematological malignancies. Among the 16 lymphomas, there was diffuse large B cell lymphoma (DLBCL) (7), follicular center cell lymphoma (1), CLL/SLL (1), NHL-no further pathological information (7), cHL (7), and NLPHL (4). Also, we identified a total of 8 leukemia in this group. These leukemia’s were ALL (4), AML (1), and leukemia – no further pathological information (3). Among these, 28/35 (80%) of FHM were “confirmed”. These 35 FHM members are 1st (14), 2nd (16), and 3rd (5) degree relatives of 31 NLPHL patients. In these 31 families, another 30 members had 32 different solid cancers.

### Families with NLPHL

There are 4 families with NLPHL in family members; all these 8 NLPHL patients are male and are alive. Family-001, age at the time of diagnosis of 2 brothers was 38, and 40 years, their sister with follicular center cell lymphoma was 36 years at diagnosis. Family-002, two brothers, 12 and 20 years at diagnosis. Family-003, 47 years and his nephew was 33 years, and Family-004, 14 years, and his 3rd degree male cousin 25 years at diagnosis.

We also checked the age at diagnosis in the same generation (25 patients) with FHM (siblings (1st degree) and cousins (3rd degree)). Age of our NLPHL patient: age of the family member with - diagnosis is shown in this format: 21:24-DLBCL, 32:28-DLBCL, 8:21-DLBCL, 21:25-cHL, 13:19-cHL, 12:20-NLPHL, 38:40-NLPHL+sister age 36-follicular center cell, 25:30-cHL, 12:10-ALL, 21:4-ALL, 18:7-ALL, and 56:80-lymphoma-no further details.

### Family information and degree of relation

Information regarding consanguinity marriages was available in 96 patients; 32/96 patients (33%) reported consanguine marriages of their parents. The median number of 1st, 2nd and 3rd degree relatives was 10 (range 3–26, 104 patients answered), 21 (range 4–54, 104 patients answered), and 50 (range 10–187, 82 patients answered; with 30 of them simply marked/said “more than a specific number”, i.e. 50+,90+, …) respectively. Median collective 1st + 2nd + 3rd degree members are 81. These 170 patients provided information about 8225 family members. Based on the median numbers of the relatives, approximately 13,700–14,000 total family member’s information is needed to be captured if all these patients can provide a detailed answer.

### Anticipation

Decrease in the age of diagnosis from 1st generation to the 2nd generation (anticipation) was studied in 17 applicable FHM patients; 13/17 showed this anticipation phenomenon. The median age of our patients (2nd generation) with NLPHL was 29.7 years vs 53 years in the parents or a 2nd degree relative (1st generation). The cancer was seen at a median of 23.3 years earlier.

### Solid cancers

Due to the reasons already explained, no “extra” and meticulous efforts were carried out confirm the reported cases. We identified 135 sold cancers in these patients and were able to confirm 33.3% cases. Detailed results are shown in Table 1 and Fig. 1.

## Discussion

We are reporting the first initiative towards a comprehensive data collection of FM and FHM from the Middle East. The data collection is ongoing and approximately (1000 /2000 cases completed). The current analysis is limited to NLPHL due to resources. This data not only showed that the FHM exists here, but the magnitude is also large and warrants a properly planned coordinated approach.

In these 170 NLPHL, we have observed 54% FM, almost 18% FHM, and even confirmed 80% of FHM and 33.3% of sold cancers too. We identified 31 families; 35 members of FHM. An interesting observation is relatively similar ages of diagnosis in many paired family members despite different hematological malignancies. Given the small sample size and early ages of diagnosis for most NLPHL, HL, and ALL, it is difficult to draw any conclusion.

We encountered many unexpected hurdles and learning experiences. Due to the large family size and time limitations of “regular clinic visit” in our current practice setup, only 50–60% of the patients filled the complete numbers of their relatives in the FM-CRF, especially cousins. Also, many patients were not fully aware of FM. Contacting patients/ their parents with the cell phone or after 5 pm from the hospital number was not very encouraging either as this was not answered much time due to an “unknown” phone number. Searching a patient’s relative treated at our institution was not easy either due to English spelling; National Identity Documents are in Arabic and English spelling was entered by hospital staff as they “decided”. It was not uncommon to have 4–8 various spelling combinations of first + last name for the computer search. Confirmation of reported diagnosis was also difficult as reported by Chang et al. [15]. Many relatives were not eager to provide any information with limited confirmation through the Saudi Cancer Registry. Not only this, in the Middle East, multiple marriages, tribal social lifestyles, consanguinity marriages in the same tribe, and issue of half-uncle and aunties and half-cousins is making it very clear that a very meticulous time-consuming custom-designed data collection in a proper pedigree software is needed for future analysis. Also, coronavirus disease − 19 pandemic in February 2020 in the Kingdom of Saudi Arabia, like the whole world, resulted in significant limitations in the staffing, verification process, and expansion of study setup that is likely to last for many more months.

There is limited literature on the familial aspect of NLPHL and other FHM [1–9]. CLL appears more common in families [4, 7]. Australian Familial Haematological Cancer Study (AFHCS) identified 24 families with apparent predisposition to hematological malignancy and suggested that at least 200 such families may exist within Australia. This was published by Carmichael C and Scott H in their society journal (not indexed) “Cancer Forum” 2007:31;160–164, “Familial aspects of haematological malignancy” (https://www.cancer.org.au). InterLymph Consortium Study, one of the largest, reviewed pooled case-control division (10,211 cases and 11,905 controls) also confirmed an increased familial risk of lymphomas.

Only a few reports are showing FM in NLPHL patients [10–14]. The largest data is from the Finnish Registry data on NLPHL and the standardized incidence ratio was 19% in the 1st degree relatives 18. They evaluated 692 patients and 4280 1st degree relatives [13]. We are likely to have almost 40% more 1st degree relatives. Giles et al. identified 13 potential families with NHL and found that the overall risk for 1st degree relatives of an affected individual was 3.15–3.61 [3].

This study provided us many important insights, both in terms of FM and FHM findings and the unique insights for planning these studies in the Middle Eastern countries in terms of interviewing technique, time and resource allocation, methods for confirming malignancies, and utilization and limitation of established national cancer registries. Once our targeted larger data is available, we will be in a better position to execute the main project of genetic counseling and genetic analysis using the latest techniques. This will be a project by itself to explain and educate accessible patients/family members about the importance of FM and involve them in our future genetic studies.

## Supplementary Information

**Additional file 1: Supplementary Data 1**. Family history of malignancy questionnaire in Arabic and the translation: For the patients

## Abbreviations

FHM: Familial clustering of lymphoid and/or hematological malignancies
NHL: Non-Hodgkin lymphoma
HL: Hodgkin lymphoma
MM: Multiple myeloma
CLL/SLL: Chronic lymphocytic leukemia/small lymphocytic lymphoma
NLPHL: Nodular lymphocyte-predominant Hodgkin lymphoma
FM-CRF: Family history of malignancy (FM) case report form
DLBCL: Diffuse large B cell lymphoma
AFHCS: Australian Familial Haematological Cancer Study
