# Supplementary material for: Risk of hematological malignancies in the families of patients treated for nodular lymphocyte-predominant Hodgkin lymphoma
Source: Hered Cancer Clin Pract. 2021 Feb 9;19:17. doi: 10.1186/s13053-021-00175-0 (PMC7871140; doi:10.1186/s13053-021-00175-0)
Supplement: Supplementary file 1 — Additional file 1: Supplementary Data 1. Family history of malignancy questionnaire in Arabic and the translation: For the patients [file 13053_2021_175_MOESM1_ESM.docx]

**رقم المريض الطبي :**

**إسم الاستشاري :**

**تاريخ الزياره :**

- **عزيزي المريض الرجاء الاجابه على الاسئله التاليه لغرض جمع بيانات تختص بالتاريخ المرضي للعائلة من ناحيه الاورام السرطانيه على أن يكون قريب كالتالي:**

**(أبنائك الأم, الأب, الأخ أو الأخت, الجد أو الجدة, العم أو العمة أو أبنائهم, الخال أو الخالة أو أبنائهم )**

- **هل لديك بالعائلة تاريخ مرضي في أورام سرطانية غير حميده ؟**
- **نعم**
- **لا**
- **اذا كان الجواب نعم وتلقى أحد العلاجات سواء سابقا أو حاليا (اذا كان القريب متوفي بسبب المرض الرجاء ذكر سنة الوفاة) :**

| **صلة القرابة** | **نوعه و موقعه** | **هل تلقى أحد العلاجات التاليه** | | | **في أي عام شُخص بالمرض** | **المستشفى الذي تلقى فيه العلاج** | **هل ما زال على قيد الحياة ؟** |
| --- | --- | --- | --- | --- | --- | --- | --- |
|  |  | **كيمياوي** | **جراحي** | **إشعاعي** |  |  |  |
|  |  | **كيمياوي** | **جراحي** | **إشعاعي** |  |  |  |
|  |  | **كيمياوي** | **جراحي** | **إشعاعي** |  |  |  |
|  |  | **كيمياوي** | **جراحي** | **إشعاعي** |  |  |  |

- **إذا كان المريض المذكور أعلاه تلقى علاجه في مستشفى الملك فيصل التخصصي ( قسم الأورام ) فأذكر ما يلي :**

| **اسم المريض الرباعي** | **رقم الملف إن وجد** |
| --- | --- |
|  |  |
|  |  |

- **هل أنت متزوج أو سبق لك الزواج؟ ( نعم / لا )**
- **هل زوجك أو زوجتك قريب لك من العائله ( أبن أو أبنة عم / عمه , أبن أو أبنة خال / خاله )؟**
- **كم عدد أبنائك ( ذكور و إناث )؟**
- **هل الأم و الأب أقرباء ( أبن أو أبنة عم / عمه , أبن أو أبنة خال / خاله )؟**
- **مجموع أخوانك الأشقاء من نفس الأم و الأب ................ مجموع أخواتك الأشقاء ..............**
- **مجموع أخوانك الغير أشقاء ................ مجموع أخواتك الغير أشقاء ........................**
- **مجموع الأعمام من جهه الأب ................ مجموع العمات ......................................**
- **مجموع أبناء العم (تقريبا) ................ مجموع أبناء العمه (تقريبا) ……….............**
- **مجموع عدد الخال من جهة الأم ................. مجموع عدد الخالة ..................................**
- **مجموع أبناء الخال (تقريبا) ................ مجموع أبناء الخاله (تقريبا).........................**

**Medical Record Number:**

**Name of the Consultant:**

**Date of the Visit:**

- **Dear patient, in order to collect the information related to family history of cancer, please answer the following questions related to your family members:**

**(Son, daughter, mother, father, brother, sister, grand fathers and mothers, uncle and aunty (from father’s side) and their sons and daughters, uncle and aunty (from mother’s side) and their sons and daughters).**

**Is there any history of a tumor of malignant nature in your family?**

- **Yes**
- **No**
- **If the answer is yes for any individual, please provide details of previous and current treatments (if anyone had died of cancer, please provide year of death).**

| Is the patient alive? | Year of diagnosis | Hospital where treatment received | What type of treatment received | | | Type and location | Relationship |
| --- | --- | --- | --- | --- | --- | --- | --- |
|  |  |  | chemotherapy | surgery | Radiation |  |  |
|  |  |  | chemotherapy | surgery | Radiation |  |  |
|  |  |  | chemotherapy | surgery | Radiation |  |  |
|  |  |  | chemotherapy | surgery | Radiation |  |  |

- **If the above patient, was he treated at KFSH&RC (Oncology Center), please provide the following information:**

| Medical Record Number: | Name of the patient (complete four names) |
| --- | --- |
|  |  |
|  |  |

- **Are you married/ever married? (Yes / No)**
- **Is your wife / husband (spouse) your cousin (either from father's or mother's side)?**
- **How many son ……….……. and daughter ……….…. you have?**
- **Is your father and mother are cousins (either from father's or mother's side)?**

- **Total number of brothers from same father and mother ……..………**
- **Total number of sisters from same father and mother ……………..**
- **Total number of stepbrothers ……………..**
- **Total number of stepsisters ……………..**
- **Total number of father’s brothers ……………..**
- **Total number of father’s sisters ……………..**
- **Total number sons and daughters of father’s brothers (approximate, if exact not known) ………………**
- **Total number sons and daughters of father’s sister (approximate, if exact not known) ……………..**
- **Total number of mother’s brothers ……………..**
- **Total number of mother’s sisters ……………..**
- **Total number sons and daughters of mother’s brothers (approximate, if exact not known) ……………..**
- **Total number sons and daughters of mother’s sister (approximate, if exact not known) ……………..**
